# Supplementary material for: The genome sequence of the Coppice Mining Bee, Andrena helvola (Linnaeus, 1758)
Source: Wellcome Open Res. 2025 Feb 24;10:102. [Version 1] doi: 10.12688/wellcomeopenres.23746.1 (PMC11897692; doi:10.12688/wellcomeopenres.23746.1)
Supplement: Peer review history

# Reviewer report: Reviewer response for version 1

**DOI:** 10.21956/wellcomeopenres.26194.r119493

**Published:** 2025-03-14

**Authors:** Pilar De la Rúa

**Affiliations:** 1 University of Murcia, Murcia, Spain

This manuscript deals with the whole genome sequencing of a mining bee with a wide Palaearctic distribution. Different technologies and platforms have been used to obtain the data.

 A doubt arises about the complementarity of the different approaches used. Has any analysis been done to compare the information obtained?

**Other small doubts are:**

- What is the rationale for using different parts of the organism for each technology? should be briefly justified
- The small dissected sample refers to a leg?

Are sufficient details of methods and materials provided to allow replication by others?

Yes

Is the rationale for creating the dataset(s) clearly described?

Yes

Are the datasets clearly presented in a useable and accessible format?

Yes

Are the protocols appropriate and is the work technically sound?

Yes

Reviewer Expertise:

population genetics

I confirm that I have read this submission and believe that I have an appropriate level of expertise to confirm that it is of an acceptable scientific standard.

# Reviewer report: Reviewer response for version 1

**DOI:** 10.21956/wellcomeopenres.26194.r120065

**Published:** 2025-03-11

**Authors:** Ricardo Rodriguez-de-la-vega

**Affiliations:** 1 Écologie Systématique et Évolution, CNRS, Université Paris-Saclay, Paris, France

Comments on Falk et al., "The genome sequence of the Coppice mining bee..."

 Another day, another genome assembly Data Note from the Darwin Tree of Life project (DToL). Long-read HiFi, chromosome-contact Hi-C, and RNAseq sequencing were performed on a single female specimen (thorax, head, and abdomen, respectively). Hifiasm assembly produced two haplotypes, with the primary being more complete than the alternate in terms of k-mer completeness and total length. Only a single BUSCO completeness score is reported, but I believe DToL genome reports for diploid organisms should include one for each haplotype. Most scaffolds are anchored to pseudo-chromosomes. Unusually for a DToL genome report, no genome annotation is provided (I had the impression this was mandatory, but after checking other *Andrena* species assemblies linked on GoaT, I confirmed this is not always the case). As is common in DToL genome assemblies, non-chromosome-anchored scaffolds are also unassigned taxonomically; I think this should be made more explicit in the main text.

Are sufficient details of methods and materials provided to allow replication by others?

Yes

Is the rationale for creating the dataset(s) clearly described?

Yes

Are the datasets clearly presented in a useable and accessible format?

Yes

Are the protocols appropriate and is the work technically sound?

Yes

Reviewer Expertise:

Genomics, Bioinformatics, Evolution

I confirm that I have read this submission and believe that I have an appropriate level of expertise to confirm that it is of an acceptable scientific standard.

## References

[1] : Genomes on a Tree (GoaT): A versatile, scalable search engine for genomic and sequencing project metadata across the eukaryotic tree of life. *Wellcome Open Res* .2023;8: 10.12688/wellcomeopenres.18658.1 24 10.12688/wellcomeopenres.18658.1 36864925 PMC9971660

# Reviewer report: Reviewer response for version 1

**DOI:** 10.21956/wellcomeopenres.26194.r120059

**Published:** 2025-03-06

**Authors:** Jason Charamis

**Affiliations:** 1 Foundation for Research and Technology - Hellas, Irákleion, Greece

This data note describes the genome sequencing of the Coppice Mining Bee, *Andrena helvola*. The work is technically sound and the produced assembly is of good quality, as demonstrated by the QV, k-mer completeness and BUSCO metrics. It provides a nice genomic resource for future ecological studies in mining bees. 

 I have only two comments to make. First and most important, the ecological relevance and rationale behind sequencing the genome of a mining bee species is not discussed in the Background section. Mining bees (Andrenidae) serve as primary plant pollinators in wild ecosystems and are considered keystone species, highly contributing to maintaining biodiversity and ecological balance. However, several of their biological and ecological attributes (specialized nesting requirements, limited flight ranges and often single annual generation cycles) increase their vulnerability to human-driven threats such as habitat destruction and pesticide exposure. In this context, sequencing the genome of a mining bee species will be a great resource for future ecological studies. Second, it would be useful to have citations for the different assembly quality metrics employed in this study.

Are sufficient details of methods and materials provided to allow replication by others?

Yes

Is the rationale for creating the dataset(s) clearly described?

Yes

Are the datasets clearly presented in a useable and accessible format?

Yes

Are the protocols appropriate and is the work technically sound?

Yes

Reviewer Expertise:

Arthropod Comparative Genomics

I confirm that I have read this submission and believe that I have an appropriate level of expertise to confirm that it is of an acceptable scientific standard.
